# Consequences for the Elderly After COVID-19 Isolation: FEaR (Frail Elderly amid Restrictions)

**DOI:** 10.3389/fpsyg.2020.565052

**Published:** 2020-09-28

**Authors:** Matteo Briguglio, Riccardo Giorgino, Bernardo Dell'Osso, Matteo Cesari, Mauro Porta, Fabrizia Lattanzio, Giuseppe Banfi, Giuseppe M. Peretti

**Affiliations:** ^1^IRCCS Orthopedic Institute Galeazzi, Scientific Direction, Milan, Italy; ^2^Residency Program in Orthopedics and Traumatology, University of Milan, Milan, Italy; ^3^Psychiatry 2 Unit, Luigi Sacco University Hospital, University of Milan, Milan, Italy; ^4^Department of Psychiatry and Behavioral Sciences, Stanford University, Stanford, California, CA, United States; ^5^Geriatric Unit, IRCCS Fondazione Ca' Granda Ospedale Maggiore Policlinico, Milan, Italy; ^6^IRCCS Orthopedic Institute Galeazzi, Movement Disorder Center, Milan, Italy; ^7^IRCCS INRCA, Scientific Direction, Ancona, Italy; ^8^Vita-Salute San Raffaele University, Faculty of Medicine and Surgery, Milan, Italy; ^9^IRCCS Orthopedic Institute Galeazzi, Regenerative and Reconstructive Unit, Milan, Italy; ^10^Department of Biomedical Sciences for Health, University of Milan, Milan, Italy

**Keywords:** frailty syndrome, coronavirus, SARS-CoV-2, COVID-19, infections, nutritional status, sedentary behavior, quality of health care

## The New Strain SARS-COV-2 Meets a Frailty Endemics

Older adults rank in the most at-risk segment of the population because the basal functional resilience, meant as the ability to cope with physical trauma and psychological stressors, is fading (Cesari et al., 2017). Aging is physiologically associated with cognitive decline and impaired stress response (Bishop et al., 2010), with the spinal circuitry degeneration leading to progressive alterations of motor performance (Borzuola et al., 2020). This reduced resilience and cognitive impairment intimately coexist in the rampant -definitely endemics- frailty syndrome (Ofori-Asenso et al., 2019), which is known to be associated with disability, traumatic falls, and hospital admission (Eeles et al., 2012). Regrettably, the wearisome settings of hospital wards provide poor incitements to the oldest minds and often oversee the abilities of individuals, who cope with progressive restlessness, dietary impoverishment, and nutrition-related or activity-related sarcopenia (Eeles et al., 2012; Ligthart-Melis et al., 2020). Multidisciplinary interventions, such as the HEPAS approach (Healthy Eating, Physical Activity, and Sleep), are models for dealing with multiple issues simultaneously (Briguglio et al., 2020c). Despite this knowledge, contemporary society, and health services put the older adults in the background. From the most complex digitization of services to the simplest use of public transport, there is “No Country for Old Men” (Ethan and Joel Coen, 2007). It is therefore not surprising that when the new strain coronavirus SARS-CoV-2 (Severe Acute Respiratory Syndrome-Coronavirus of 2019) spilled out to infect humans found not only fertile ground -a population of old people- but also countries ready to choose treating people with more life expectancy. After the outbreak of viral pneumonia in Wuhan, China, (December 2019), SARS-CoV-2 spread rapidly in Europe. Italy resulted among the worst-hit countries with 214.457 infected and 29.684 deaths (May 7, 2020, WHO situation report 108). The northern region of Lombardy accounted for the overall 52.3% of the deaths (May 7, 2020, Italian SARS-CoV-2 Surveillance Group), with the older adults suffering from chronic cardiovascular diseases and malnutrition counting the highest case-fatality ratio (Briguglio et al., 2020b). Considering that the region counted 128.528 subjects over 60 years of age at the beginning of the past year (Annual Italian Census of 2019), we can say that the north of Italy lost over 10% of its older population. This rapid increase of infected severe cases led to a rapid saturation of health facilities in March-April 2020 and public health interventions focused on social isolation, travel restraints, and at-home confinement. Containment measures have been applied with different degrees of restriction in different Italian regions, but the northern regions -the worst-hit- have suffered the most severe lockdown measures. In Lombardy, almost 100.000 older adult residents locked themselves up in the house.

## Public Health Interventions Amid COVID-19: Locking up in Fear Pandemic

Leaving the house was permitted, but only for proven health or job reasons. Interregional travel was also banned. Most commercial activities were shut down, few have been minimized. Buying necessities was allowed, but only one individual per family wearing masks and gloves. To respect social distancing, supermarkets regulated the entrances eventually forming long queues, with people possibly waiting for hours. Priority tickets could be booked online, as well as masks that were sold out by pharmacies but available on various web sites at inflated prices. Eventually, these measures contributed to reduce the impact on health services and the risk of severe illness (Steffens, 2020). Although reasonable and essential, the social lockdown has affected both the bourgeois and the less well-off classes of the population. However, are the vulnerable groups -the older adults- who will be carrying the worse future debt of disability? In the pre-COVID-19 era, over 50% of older adults were known to be at risk of loneliness (with associated morbid events) (Fakoya et al., 2020) and this feature fused with reduced health care capacity during the pandemic. In the COVID-19 era, most medical clinics closed or adhered to special hours and the reorganization of the health system led to a significant reduction in clinical and surgical assistance. These restrictions prevented the older adults from having a continuity of care for their co-existing chronic conditions. The decline in social relations combined with reduced support increase the disability debt, with the reaching of the “social frailty.” Results from a Chinese -another worst-hit country- online survey proved over 50% of respondents rating the psychological impact of COVID-19 moderate-to-severe, with depressive and anxiety symptoms being prevalent (Wang et al., 2020). Dramatic events, such as the loss of a kin, but also anxiety from the fear of being infected and the inability to do something can further compromise the mental health. On one hand, the Italian daily newscast informed the public about the disease severity, reporting hundreds of daily deaths. On the other hand, the indirect fear inherent in those who were watching has been a major side effect. Frailty therefore acquired a mental nature, becoming “psychological frailty” (Gobbens et al., 2012). Older adults require increasing cognitive demand to perform any motor task (Seidler et al., 2010). The COVID-19 restrictions have been not only associated with psychological derangements, but also with an increasing “bed-kitchen-sofa” lifestyle. Low environmental information-processing was consequently prevalent during daytime, with further impairment of age-associated spatial disorientation, proprioception, disequilibrium, and incoordination (Dunsky, 2019). At-home confinement easily led to sarcopenia. The sedentary lifestyle associated with constant stress that decreased the desire to eat. Either reduced food security or food supply reduced energy intake, leading to nutritional deficits (Briguglio et al., 2020b). Sarcopenia easily became osteosarcopenia. After 2 months (end of March, April, and early May) of confinement, the perceived loss of balance inherits the fear of falling. The “physical frailty” reaches its peak.

## End of Isolation: Looking up for Fear Consequences

The easing of COVID-19 lockdown on the older population has possibly brought effects comparable to the hospital-associated deconditioning. The disability debt earned during the lockdown will require an augmented need for care for older individuals suffering from the abovementioned geriatric conditions -functional disability- and psychosocial disorders, mainly isolation. The surviving older individuals who have not been infected with SARS-CoV-2 are definitely more fragile, malnourished, and more ill than the pre-COVID-19 era. Those who have been infected will encounter permanent disabilities, such as pulmonary fibrosis and impaired liver function. Indeed, reduced respiratory capacity has been observed for the survivors after SARS-CoV-1 (Ngai et al., 2010). Permanent affections could be also mental, with long-term neuropsychiatric consequences being characteristics of neurotrophic coronaviruses (Briguglio et al., 2020a; De Felice et al., 2020). We expect the frail older adults to be exposed to an increased risk of traumatic events amid restrictions (Clegg et al., 2013). This worsening of the three-dimension frailty may therefore transduce into more hospital admissions. Frail older adults encounter a 1.2- to 2.8-fold risk for falls and fractures and 1.2- to 1.8-fold risk for hospitalization (Vermeiren et al., 2016). Even though COVID-19-associated admissions are known to be flattening, it is also known that most fractures occur in the home and the prolonged restrictions may expose orthopedic hospitals to a different kind of saturation post-COVID-19. During the pandemic, the choice of operating older adult subjects who have suffered a falling trauma was a matter of debate. In the worst-hit countries, the experience of Chinese (Mi et al., 2020) and Spanish (Munoz Vives et al., 2020) authors would suggest delaying the surgical treatment of fractured patients with SARS-CoV-2 as they have observed excessive mortality rates. The Italian experience would suggest instead to treating the fracture as soon as possible in order to stabilize the patient (Catellani et al., 2020). Anyhow, it is a fact that elective orthopedic surgery has been delayed, but there may be also a debt of traumatic fractures that must be bridged. It is also possible that many lonely seniors who fell into the house during the restricted period have not yet been established: fall not reported? While there was over a halving of emergency room accesses for high energy fractures in Italy during the pandemic (Fojut, 2020; Magro et al., 2020), on the other hand, low energy/fragility fractures did not substantially reduce (Benazzo et al., 2020; Jain et al., 2020). This highlights the lack of home prevention measures for the elderly that certainly has exposed them to an increased risk of risk of hospital-acquired SARS-CoV-2 infection.

## Discussion

**Figure 1:**
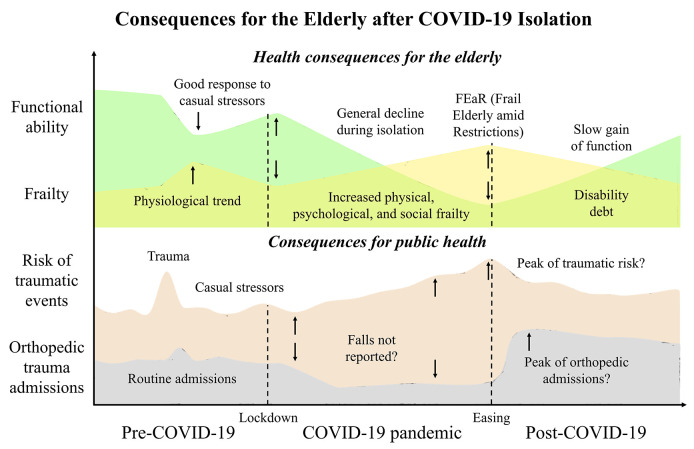
Tendencies of the consequences of the COVID-19 pandemic on the health of the elderly and the risk of hospital admissions after lockdown easing. The severe acute respiratory syndrome coronavirus that was discovered in Hubei province, China at the end of December 2019 (SARS-CoV-2) burst a pandemic that shut down the world. Under normal circumstances, the older adults must deal with a diminished functional ability and progressive establishment of geriatric fragility. If a healthy status is present, recovery from any trauma is discreet even if it is not associated with full pre-trauma functionality. Although restrictive measures were necessary for public health during the pandemic, they have exposed older individuals to confinement that has worsened their physical and mental health. Upon easing of the lockdown, the older person will suffer a disability debt, which will make him highly vulnerable to the risk of falling. Orthopedic hospitals that recorded a halving of traumatic admissions could contrariwise encounter a surge in accesses for traumas amid lockdown easing.

In the post-COVID-19 era, the saturation of health services may only be the tip of the iceberg in relation to the restriction-derived burden of frailty. During confinement, the diminished state of resilience in elderly people may have worsened all age-associated conditions, such as a mild high blood pressure, glucose intolerance, basal immune dysfunction, inflammaging, and mental liability with anxiety-depressive traits. The dynamics of “frailty” renders its transition to a worse level more common than improvement (Morley et al., 2013), and this COVID-19 pandemic may have spin the loop of a decline of decreasing functional ability, increasing frailty, greater risk of traumatic falls, and higher hospital admissions for fragility fractures in the near future (Figure 1). Since it is difficult to predict when SARS-CoV-2 will become a secondary problem, it is also important to ponder the possibility of a second wave of infections since this prolonged social isolation has created a population with fewer anti-viral immune defenses (Cole et al., 2015). Homeless and people with disabilities should also be a matter of concern (Mesa Vieira et al., 2020). It is therefore mandatory to get prepared for pandemic consequences with appropriate interventions, being both public health-oriented and patient-oriented. This pandemic has not only underlined the public health challenges to guarantee that older population can access the services they need, but it has also shown new opportunities to be seized, such as an expanded workforce specialized in aging (Morrow-Howell et al., 2020), a promotion of intergenerational solidarity (Brooke and Jackson, 2020), or practical community participations, such as the dropping off of groceries (Fraser et al., 2020). In clinical settings, the confronting with a fast-growing geriatric population suffering from multiple comorbidities needs a multidisciplinary approach like the orthogeriatric co-management model of care, with orthopedic doctors and geriatricians prioritizing the patients' needs and aiming at clinical as well as cost-benefit advantages for older adults (Gosch et al., 2016). Valuable and tailored patient-oriented solutions have been proposed after COVID-19 pandemic restriction-associated isolation to cope with social, psychological, and physical frailty. Both the procurement of health care assistance and the reduction of loneliness to older adults that who have suffered from isolation should be a priority for social frailty handling. For instance, remote interventions via online systems may be valuable (Patel and Clark-Ginsberg, 2020) but an appropriate utility assessment and training in the use of technological services should be provided. Psychological frailty should be counteracted through older adult engagement and motivation, possibly via phone contact with health professionals (Armitage and Nellums, 2020) or by broadcasting television entertaining with premeditated programs (Jawaid, 2020). Also, symptoms such as fear and sleeping disturbances should be properly identified and addressed (Berg-Weger and Morley, 2020). Physical frailty can be resolved through educational videos and recorded physical activity sessions (Angulo et al., 2020). Actually, online technologies are the most valuable support systems, but have to be appropriately planned for older minds (Meinert et al., 2020). Of note, prioritized interventions should be established for low and middle income countries where family dynamics are different, a large number of older adults are illiterate, and proper health care assistance is limited (Lloyd-Sherlock et al., 2020).

To conclude, we can say that:

- The new coronavirus SARS-CoV-2 met a population of frail elderly
- The restrictions due to the COVID-19 pandemic generated a more fragile class of older adults
- The health system should re-organize for efficiently managing the surge of frailty fractures
- Long-term psychological consequences amid COVID-19 pandemic and associated restrictions should be considered, especially for the oldest fragile minds.

## Author Contributions

MB formulated the hypothesis and wrote the first draft of the manuscript together with RG. BD, MC, MP, FL, GB, and GMP revised the first draft and contributed to manuscript sections. All authors contributed to manuscript revision, read and approved the submitted version.

## Conflict of Interest

The authors declare that the research was conducted in the absence of any commercial or financial relationships that could be construed as a potential conflict of interest.
